# Comparison of the Nutritional Properties and Transcriptome Profiling Between the Two Different Harvesting Periods of *Auricularia polytricha*

**DOI:** 10.3389/fnut.2021.771757

**Published:** 2021-10-26

**Authors:** Wenliang Wang, Yansheng Wang, Zhiqing Gong, Shifa Yang, Fengjuan Jia

**Affiliations:** ^1^Shandong Academy of Agricultural Science, Jinan, China; ^2^Institute of Agro-Food Science and Technology, Shandong Academy of Agricultural Sciences, Jinan, China; ^3^Key Laboratory of Agro-Products Processing Technology of Shandong Province, Jinan, China; ^4^Key Laboratory of Novel Food Resources Processing, Ministry of Agriculture and Rural Affairs, Jinan, China

**Keywords:** *Auricularia polytricha*, nutritional analysis, transcriptome analysis, developmental regulation, synthesis and metabolism

## Abstract

*Auricularia polytricha* (*A. polytricha*), regarded as an edible and medical mushroom, has attracted toward the research interests because of the high nutrition and bioactivity. The nutritional and medical properties of *A. polytricha* have been well-studied; however, research about the difference of the nutritional properties and transcriptome profiling between the two different harvesting periods of *A. polytricha* was limited. In this study, the nutritional properties and transcriptome profiling were compared between the two different harvesting periods of *A. polytricha*: AP_S1 (the stage for the first harvesting period) and AP_S2 (the stage for the third harvesting period). This study showed that AP_S1 had the more growth advantages than AP_S2 including biomass, auricle area and thickness, protein and calcium contents, and most species of the amino acid contents, which contributed to the higher sensory evaluation and acceptability of AP_S1. Transcriptome profiling showed that a total of 30,298 unigenes were successfully annotated in the two different harvesting periods of *A. polytricha*. At a threshold of two-fold change, 1,415 and 3,213 unigenes were up- and downregulated, respectively. All the differentially expressed genes (DEGs) analysis showed that the some synthesis and metabolic processes were strengthened in AP_S1, especially the synthesis and metabolism of the amino acids and protein. The enhanced energy metabolism pathways could provide more energy for AP_S1 to synthesize the nutritional substance. Moreover, the expressions of 10 selected DEGs involved in the amino acid and protein synthesis pathways and energy metabolism pathways were higher in AP_S1 compared to AP_S2, consistent with Illumina analysis. To the best of our knowledge, this is the first study that compares the nutritional properties and transcriptome profiling between the two different harvesting periods of *A. polytricha* and the results can present insights into the growth and genetic characteristics of *A. polytricha*.

## Introduction

Nowadays, mushrooms have become more and more attractive because of their excellent taste and health-promoting traits (1). *Auricularia polytricha* (*A. polytricha*), belonging to the *Basidiomycota, Agaricomycetes, Incertae sedis, Auriculariales, Auriculariaceae, Auricularia*, has attracted toward the research interests because of the high nutrition and bioactivity. *A. polytricha*, regarded as an edible and medical mushroom, is widely cultivated worldwide and ranks the fourth cultivated black wood-ear fungus (2). Its secondary metabolites have the various chemical species and bioactivities (3). The fruiting bodies or powdered mycelia can be used as an alternative biosorbent to aid in the detoxification of effluents contaminated by the metals and emulsified oil (4, 5). Better insight into the regulation mechanisms underlying growth and development of *A. polytricha* should affect the commercial production of *A. polytricha* and its potential value.

During the past 20 years, various multi-omics-based studies have revealed the various details on growth, development, nutrition, metabolism, and disease resistance during the growth process of an individual (6, 7). Bioinformatics and high-throughput sequencing analysis have represented a useful approach to identify the potential genes or proteins regulating the growth and development (8). Of note, the high-throughput sequencing analysis of RNA transcripts [RNA sequencing (RNA-seq)] has become an indispensable technology for studying the transcriptome at the single-nucleotide resolution (9). Whole transcriptome analysis can quantify the gene expression differences in the different tissues and organs through the capturing coding and non-coding RNA information; this technology is also helpful for the annotation and function analysis of the genes/genomes (10). The availability of the transcriptome analysis will likely to accelerate our understanding of the development of regulation at the molecular, metabolic, and physiological levels.

At present, high-throughput sequencing analysis technology can facilitate the systematic monitoring of *A. polytricha*. Illumina Solexa sequencing technology has been used to generate very large transcript sequences from the mycelium and mature fruiting body of *A. polytricha* for the gene discovery and molecular marker development (11). The transcriptome can provide an important sequence resource to facilitate the further studies on the functional genomics and genetics of *A. polytricha*. Moreover, proteomic information of the fruiting-body proteins by the shotgun liquid chromatography with tandem mass spectrometry (LC-MS/MS) has been investigated and this is the first study to characterize *A. polytricha* proteome and has filled the gap of our knowledge on the underdeveloped mushroom species (12). In a large-scale mushroom production, cultivation season of *A. polytricha* is usually from April to October every year. *A. polytricha* has the different developmental stages to harvest for the edible food including AP_S1 (the stage for the first harvesting period) and AP_S2 (the stage for the third harvesting period) and AP_S1 of *A. polytricha* is commonly considered with the best characteristics such as high yield, good provenance, and rich nutrition (13). Since some omics datasets have provided information regarding understanding the development and regulation of *A. polytricha*, the difference in the comparison of the nutritional properties and transcriptome profiling between the two different harvesting periods of *A. polytricha* is relatively limited.

In this study, we performed a transcriptome profiling of the fruiting bodies of *A. polytricha* in the two different harvesting periods (AP_S1 and AP_S2) with the aim to understand the regulatory genes and network associated with biosynthesis, growth, and development. To the best of our knowledge, this is the first study to analyze the transcription differences of the two different harvesting periods of *A. polytricha*, which can provide the insights into the developmental process and nutritional substance changes in *A. polytricha*.

## Materials and Methods

### Materials

In this study, the fruiting bodies of *A. polytricha* were obtained in the Yutai County, Shandong province, China. The fresh bodies of *A. polytricha* were washed thoroughly, drained at the room temperature, and then oven-dried to a constant weight. After that, the dried samples were grinded into the small particles by grinder and sifted through 100-mesh sieve to eliminate the residues, getting the fine *Auricularia polytricha* powder (APP) for the further nutrition detection. In addition, all the chemicals were of analytical grade and were bought from the local chemical suppliers.

### Sensory Evaluation

To evaluate the overall acceptability of *A. polytricha* coded with a number, a total of 20 consumers aged 20–40 years old, including 10 females and 10 males, were randomly selected to carry out the sensory evaluation. Based on a nine-point hedonic scale, the hedonic test was conducted and the evaluation standards were as follows: 1 = dislike extremely, 5 = neither like nor dislike, and 9 = like extremely. During the evaluation process, the consumers used tap water to rinse mouth.

### RNA Sequencing

RNeasy Plant Mini Kit (Qiagen, Hilden, Germany) was employed to extract the total RNA and the quantification of RNA quality was conducted by using the NanoDrop 2000 (Thermo Fisher Scientific, Massachusetts, USA) and the Qubit 2.0 (Invitrogen, California, USA). The evaluation of RNA integrity was performed by using the Agilent 2100 Bioanalyzer (Agilent Technologies, California, USA). When the RNA Integrity Number (RIN) was more than 8, RNA-seq was carried out. The TruSeq RNA Sample Preparation Kit (Illumina, San Diego, California, USA) was used to construct the complementary DNA (cDNA) library. Paired-end sequencing was done by using the Illumina sequencing platform (HiSeq^TM^ 4000) (Illumina, San Diego, California, USA). The accession number for this RNA-seq data submitted to the National Center for Biotechnology Information (NCBI) was PRJNA760980.

### Transcriptome Profiling

The quality of raw reads was evaluated by using FastQC (version 0.11.9, http://www.bioinformatics.babraham.ac.uk/projects/fastqc/). Trimmomatic (version 0.39, http://www.usadellab.org/cms/?page=trimmomatic) (14) was used to trim the adaptors and low-quality bases. When the Phred quality scores of reads were more than 30, transcriptome assembly was conducted. Trinity (version 2.4.0, https://github.com/trinityrnaseq/trinityrnaseq/wiki) was employed de novo to assemble the clean reads to generate the unigenes with a minimum contig length of 200 bp and then CORSET (version 1.09, https://code.google.com/p/corset-project/) was used to remove the redundancy sequences and further spliced the longest unigenes (15). TransDecoder (version 5.5.0, https://github.com/TransDecoder) was used to extract the possible open reading frames (ORFs) and translated the unigenes to the protein sequences. After that, the annotation of unigenes, which have the coding ability, was carried out against the NCBI non-redundant (NR) protein database, the Kyoto Encyclopedia of Genes and Genomes (KEGG), and the protein family (PFAM) databases via the Basic Local Alignment Search Tool Protein (BLASTP) (version 2.10.1+, E-value < 1e-5, https://blast.ncbi.nlm.nih.gov/Blast.cgi), the KofamScan (version 1.3.0, https://github.com/takaram/kofam_scan), and the biosequence analysis using profile hidden Markov models (HMMER) (version 3.3.2), respectively. The Gene Ontology (GO) annotation was carried out by the BLASTP (version 2.10.1+, E-value < 1e-3) against the Swiss-Prot database and the GO terms of each unigene were obtained from the corresponding Swiss-Prot entries.

The Bowtie (version 1.3.0, http://bowtie-bio.sourceforge.net/index.shtml) was used to map the clean reads to the unigenes with the default parameters. The RNA sequencing by expectation-maximization (RSEM) (version 1.3.0, http://deweylab.github.io/RSEM/) was employed to estimate the abundance of the unigenes [trimmed mean of M-values (TMM)] (16). The DEseq2 (version 1.6.3, https://bioconductor.org/packages/release/bioc/html/DESeq2.html) was used to analyze the differentially expressed unigenes (DEGs) (17). When *p* < 0.05 and fold change > 2, a unigene was regarded as differential expression. The topGO package (https://www.bioconductor.org/packages/release/bioc/html/topGO.html) and the KEGG Orthology-based Annotation System (KOBAS) (version 3.0, http://kobas.cbi.pku.edu.cn/kobas3/) were, respectively, employed to perform the GO and KEGG function enrichment analyses for the DEGs (18). The gene set enrichment analysis (GSEA) was implemented on the Java GSEA platform (version 4.1.0, https://www.gsea-msigdb.org/gsea/index.jsp).

### Real-Time RT-PCR Analysis

Real-time reverse transcription-PCR (RT-PCR) was used to test the expression levels of selected genes of the fruiting bodies of AP_S1 and AP_S2. The 10 selected DEGs were involved in the amino acid and protein synthesis pathways and energy metabolism pathways. The quantitative RT-PCR (qRT-PCR) experiment was performed by using the Maxima SYBR Green qPCR Master Mix (Thermo Fisher Scientific) Kit and was completed on the Applied Biosystems 7500 fast real-time PCR system. The data are presented after normalizing to the reference gene *actin* and the three biological replicates under similar conditions were performed for each experiment. The 2^−ΔΔCt^ method was used to calculate the expression of differential genes. The primers used in this study were displayed in Supplementary Table 11 by using Primer Premier 5.0 software.

### Statistical Analysis

One-way ANOVA and Duncan's multiple comparison test were used to analyze the SD and significant differences by using with SPSS software. The data obtained were expressed as mean ± SD. A *p* < 0.05 was considered as statistically significant.

## Results

### Biological Characteristics Difference Between the Two Different Harvesting Periods of *A. polytricha*

**Figure 1:**
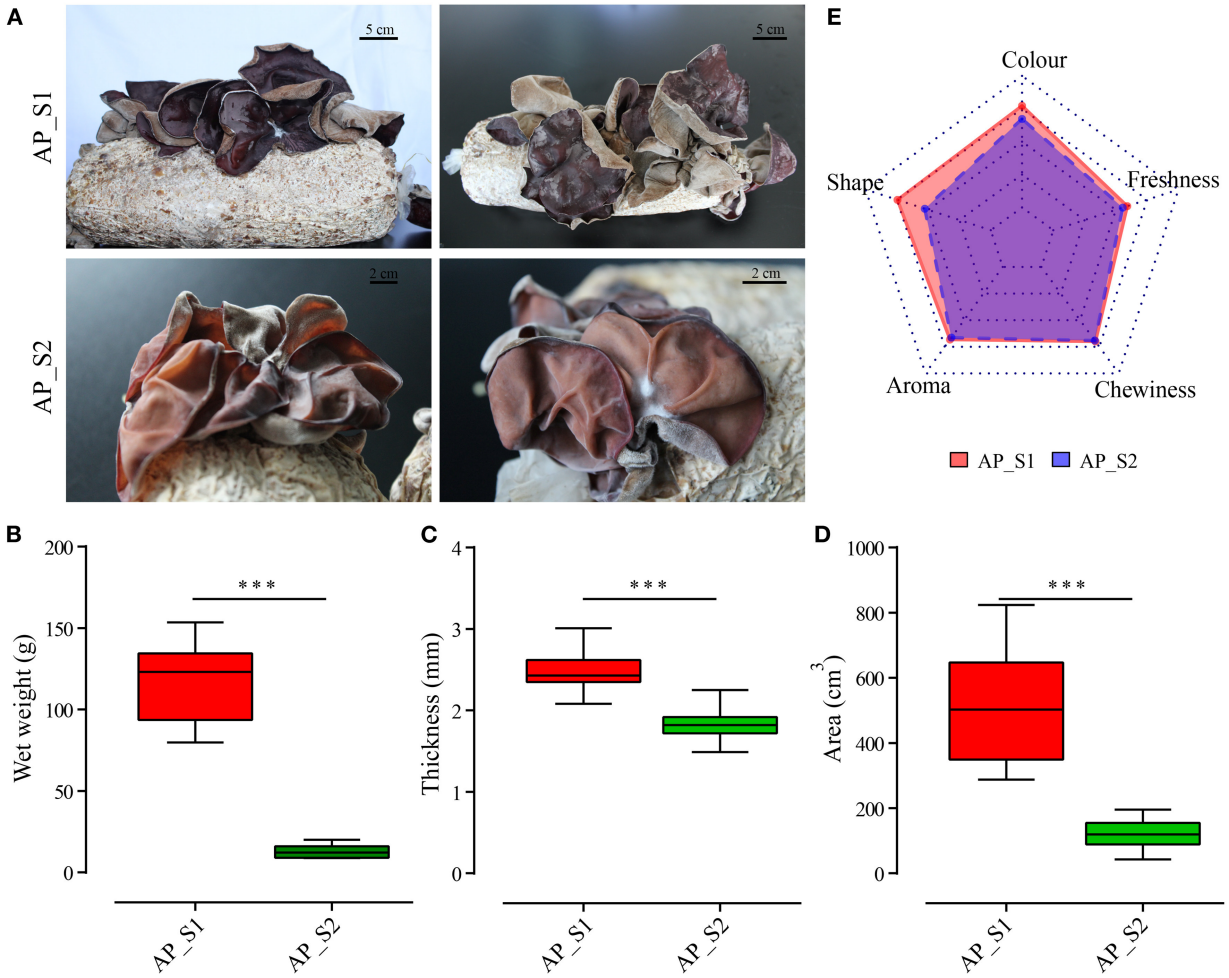
Biological characteristics and nutritional analysis between the two different harvesting periods of *Auricularia polytricha (A. polytricha)*. **(A)** Phenotype of the fruiting body of “AP_S1” (a, b) and “AP_S2” (c, d). The biomass **(B)**, area **(C)**, and auricle thickness **(D)** of “AP_S1” (red) and “AP_S2” (green), respectively. **(E)** The sensory evaluations of “AP_S1” and “AP_S2”. ***P < 0.001.

To compare the nutritional properties and transcriptome profiling between the two different harvesting periods of *A. polytricha*, the biological characteristics of two samples, namely, AP_S1 (the stage for the first harvesting period) and AP_S2 (the stage for the third harvesting period) were firstly determined. As shown in Figure 1A, we found that the fruiting body of AP_S1 was obviously larger than that of the fruiting body of AP_S2 at the mature stage. AP_S2 exhibited obviously downward-curled auricle with the more dwarfism and growth retardation. In comparison to AP_S1, AP_S2 also had the more relative fisted and thin fruiting bodies. Moreover, the color of AP_S1 was dark brown or purple, with luster, and a uniform gray tomentum behind the ears. The color of AP_S2 was light brown or purple with white or brown tomentum on the back. To further quantify the difference between AP_S1 and AP_S2, the biomass, area, and auricle thickness were measured. The results showed that the biomass of AP_S1 was five to six times higher compared to AP_S2 (Figure 1B). The auricle thickness of AP_S1 varied from 2.4 to 3.2 mm, while the thickness of “AP_S2” was only 1.5 to 2.3 mm (Figure 1C). We also found that the areas of AP_S1 were almost three to five times of AP_S2 ranging from 450 to 710 cm^2^ (Figure 1D). These results demonstrated that the AP_S1 of *A. polytricha* had the more growth advantage than the AP_S2 of *A. polytricha*.

### Nutritional Analysis Between the Two Different Harvesting Periods of *A. polytricha*

The nutrients, mineral elements, and contents of amino acids in the two different harvesting periods of *A. polytricha* were determined (Supplementary Tables 1, 2). The results showed that some of the nutrients and amino acids of AP_S1 were higher compared to AP_S2, which was consistent with the growth advantage of AP_S1. For example, the protein and calcium contents in AP_S1 were apparently higher compared to AP_S2. Levels of Fe and Zn were accumulated more in AP_S2 and the fat content of AP_S2 was also higher compared to AP_S1. The amino acid contents in AP_S1 were higher compared toAP_S2, except Met, Thr, Asp, and Phe. The total amino acid content in AP_S1 was 11.51%, while the total amino acid content in AP_S2 was 10.74%. Moreover, the sensory evaluations which included auricle size, auricle color, auricle shape, aroma, and chewiness of these two period samples were made by 20 consumers to evaluate the overall acceptability of AP_S1 and AP_S2. As displayed by a radar chart in Figure 1E, the overall sensory score of AP_S1 was significantly higher compared to AP_S2, especially the auricle shape, color, and degree of freshness. The scores of aroma and chewiness of AP_S1 and AP_S2 had no significant difference. These results indicated that the acceptability of AP_S1 was higher compared to AP_S2, including nutrition, quality, and sensory.

### Illumina Sequencing and Sequence Assembly

**Figure 2:**
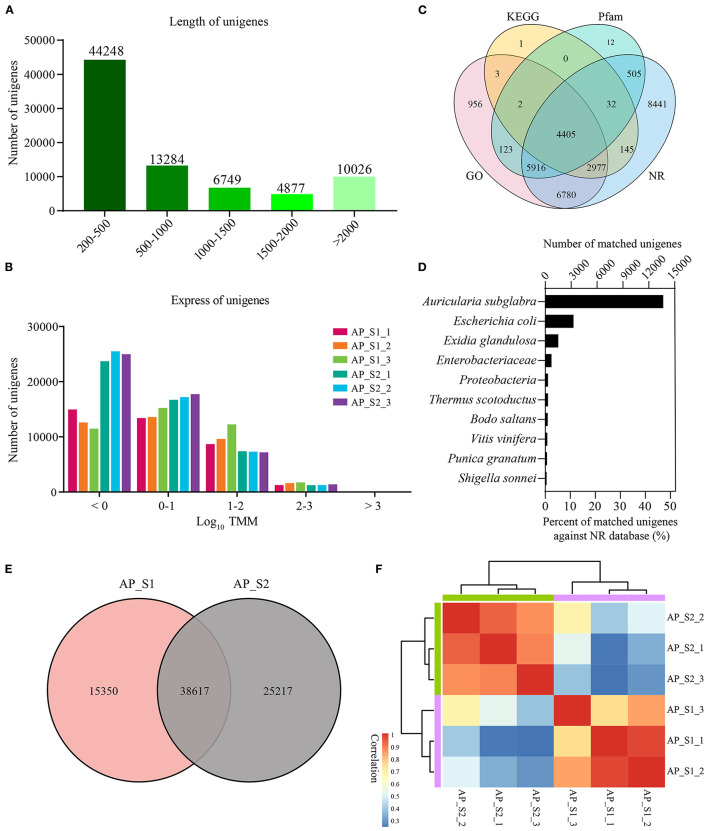
Overall results for the Illumina sequencing. **(A)** Distribution of the unigenes of different lengths. **(B)** Expression level of the unigenes among the two different harvesting periods of *A. polytricha*. **(C)** Venn diagram for the different functional annotations of the *A. polytricha* unigenes. **(D)** Top-hit species distribution of the *A. polytricha* unigenes based on the non-redundant (NR) annotation. **(E)** Venn diagram showing the number of coexpressed unigenes in “AP_S1” and “AP_S2”. **(F)** Heatmap of correlation between all the samples.

For the purpose of generating an overview of *A. polytricha* unigene expression profile of the different samples, RNA was extracted from AP_S1 and AP_S2, respectively. Each sample was conducted with three independent experiments, which was labeled as AP_S1_1, AP_S1_2, AP_S1_3, AP_S2_1, AP_S2_2, and AP_S2_3. High-throughput sequencing analysis was carried out by using the Illumina HiSeq 4000 sequencing platform. Of the average, total 68 and 109 million reads generated from AP_S1 and AP_S2. After quality filtering, approximately 60 and 100 million clean paired-end sequence reads were, respectively, obtained from AP_S1 and AP_S2 with the Q30 percentage over 90% (Supplementary Table 3). Finally, 160 million high-quality reads were assembled into 79,184 unigenes. The length of unigenes was shown in Figure 2A and the unigene size distribution showed that more than 50% of the unigenes (69,158; 87.3%) were between 200 and 2,000 bp in length. The number of unigene size with more than 2,000 bp was 10,026 and the number of unigenes in 200–500 bp was the largest with 44,248 (55.9%). The expression level of those unigenes in *A. polytricha* was shown in Figure 2B. TMM value of unigenes was mainly in the range of 0 to 100 (log_10_ TMM: 0–2) and AP_S2 has more lower expressed unigenes (log_10_ TMM < 0) compared to AP_S1. A total of 30,298 unigenes were successfully annotated against the GO (21,162), the KEGG (7,565), the Pfam (10,995), and the NR (29,201) databases, among which 4,405 unigenes were shared in the four databases (Figure 2C). The species with the highest percentage of top BLASTP hits in the NR database was *Auricularia subglabra*, accounting for 47.13% (13,754) (Figure 2D). Among the unigenes, there were 38,617 unigenes coexpressed in AP_S1 and AP_S2 (Figure 2E) and 15,350 and 25,217 unigenes were expressed specifically for AP_S1 and AP_S2, respectively. In addition, the sample correlation analysis indicated that there was a good correlation between AP_S1 and AP_S2 groups, suggesting that the Illumina sequencing and sequence assembly were reliable and available (Figure 2F; Supplementary Table 4).

### Variations in Gene Expression Between the Two Different Harvesting Periods of *A. polytricha*

**Figure 3:**
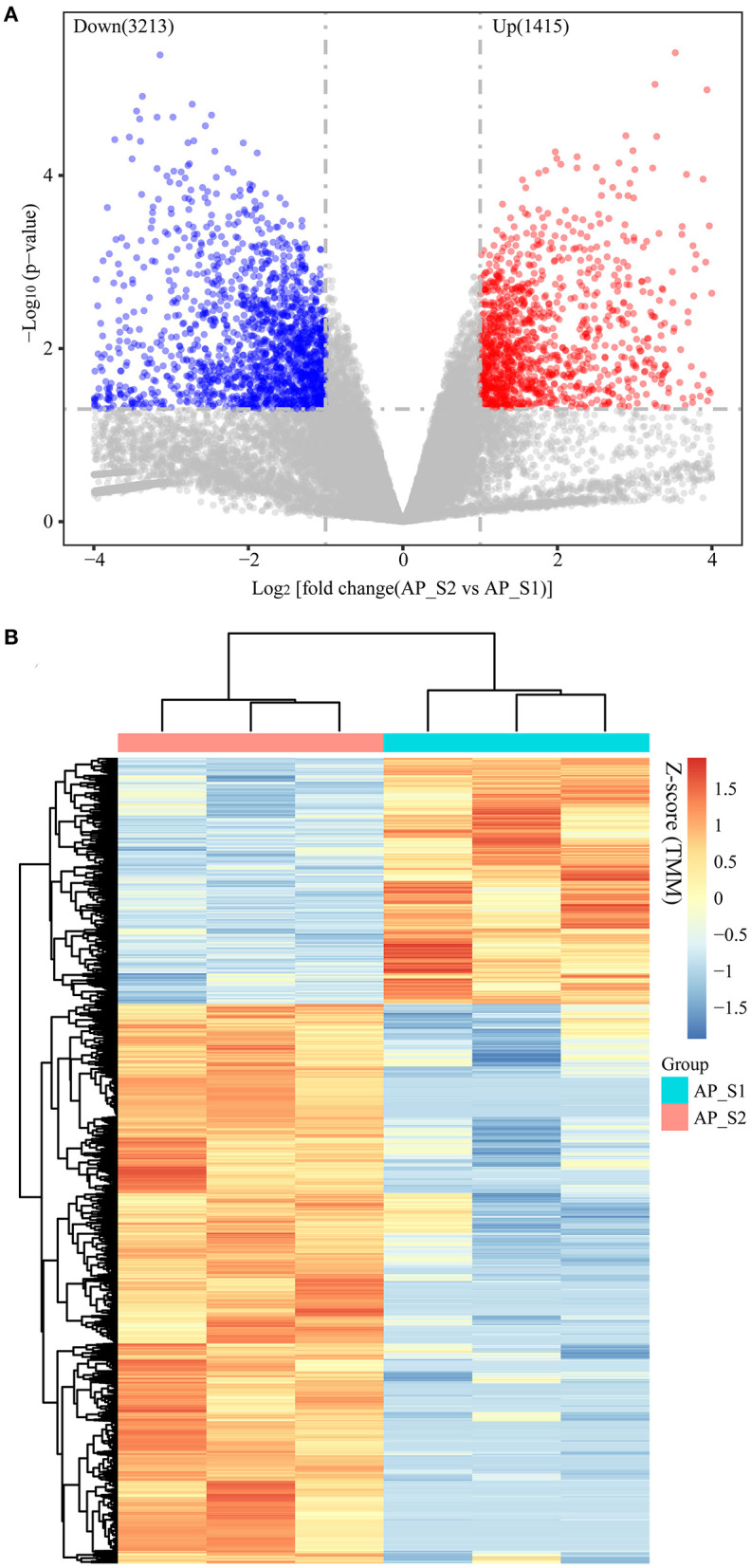
Distribution of the differentially expressed genes (DEGs) in the two different harvesting periods of *A. polytricha*. **(A)** Volcano plot of the DEGs in the two different harvesting periods of *A. polytricha*. The horizontal axis shows the log2-fold change of unigenes between the two periods. The -log10 (*p*-value) is plotted on the vertical axis. Each unigene is represented by one point on the graph. **(B)** Heatmap of the DEGs in the two different harvesting periods of *A. polytricha* expressed as *z*-score of the trimmed mean of M-values (TMM).

A total of 79,184 unigenes were identified and quantitated in the two different harvesting periods of *A. polytricha* covering the diverse metabolic and signaling pathways. Among the unigenes, under the threshold of two-fold change and *p*-value <0.05, 1,415 and 3,213 unigenes were up- and downregulated with AP_S2 vs. AP_S1, respectively (Figure 3; Supplementary Table 5). Interestingly, the number of downregulated genes was higher compared to the upregulated genes, demonstrating that the transcriptional levels of many genes were reduced in the growth and development process of *A. polytricha*.

**Figure 4:**
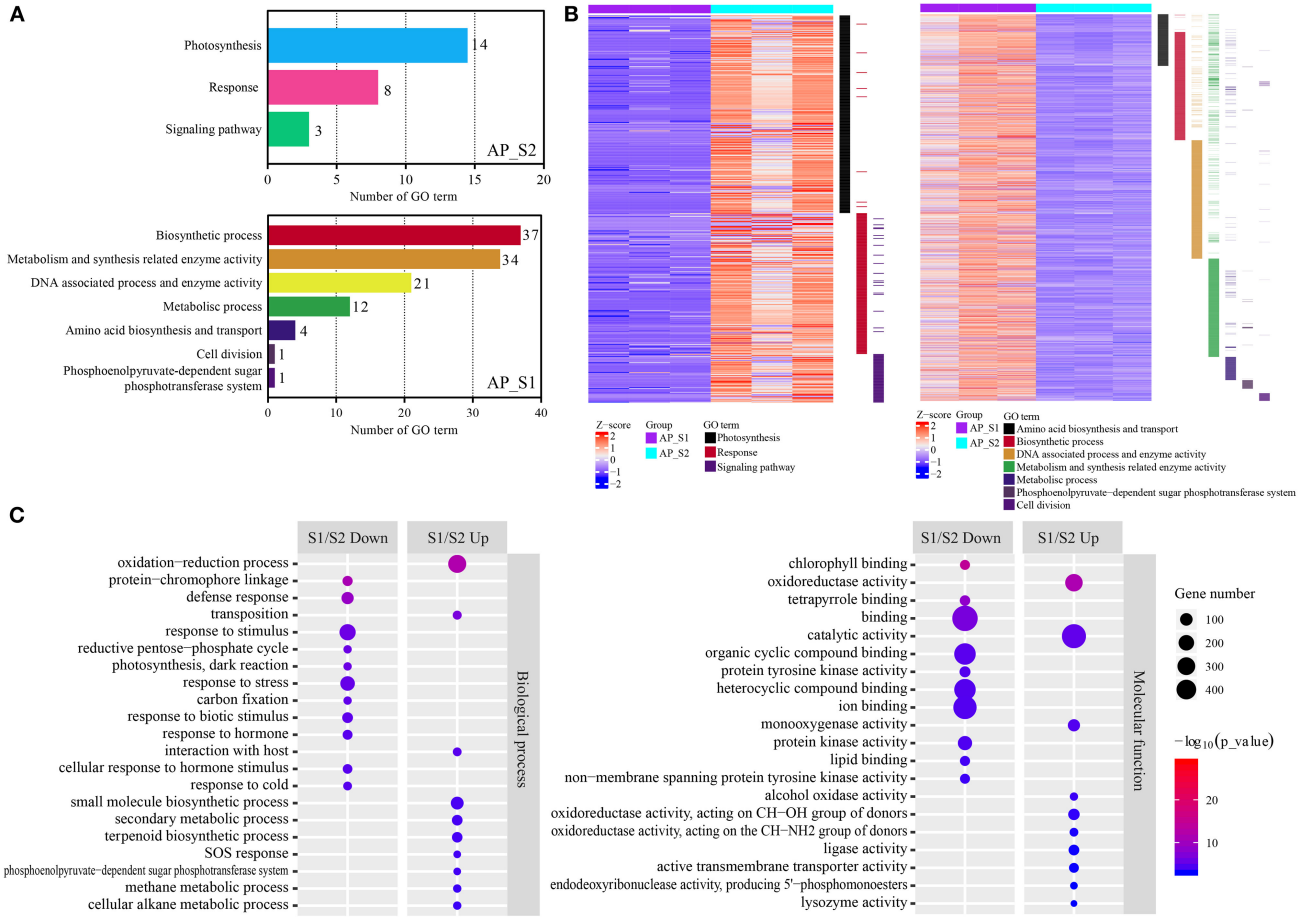
The Gene Ontology (GO) analysis between the two different harvesting periods of *A. polytricha*. **(A)** The GO analysis by the gene set enrichment analysis (GSEA) of all the unigenes. **(B)** The unigenes expression level of “AP_S1” and “AP_S2” involved in those GO terms. **(C)** Biological process (left) and molecular function (right) by the GO analysis of the DEGs.

In order to compare the biological functions and the signal pathways which involved the unigenes between the two periods of *A. polytricha*, the GO and the KEGG enrichment were analyzed by the two methods, which based on all the unigenes and the DEGs. The GO analysis of all the unigenes by the GSEA showed that the terms of “biosynthetic process,” “metabolism- and synthesis-related enzyme activity,” and “DNA-associated process and enzyme activity” were dominant in AP_S1 and the terms of “photosynthesis,” “response,” and “signaling pathway” were dominant in AP_S2 (Figures 4A,B; Supplementary Table 6). In comparison to AP_S2, the two-fold upregulated unigenes in AP_S1 were clustered to the constructive metabolism (top 10 GO terms by *p*-value) which analyzed by the topGO based on the upregulated unigenes, consistent with the GSEA analysis based on all the unigenes (Figure 4C; Supplementary Table 7). For example, the GO term analysis of “Biological process” revealed that the metabolic process such as “cellular alkane metabolic process” (42.9%), “terpenoid biosynthetic process” (16.9%), and “secondary metabolic process” (16.7%) had the highest proportion of upregulated unigenes in AP_S1, except for the high proportion of “phosphoenolpyruvate-dependent sugar phosphotransferase system” (80.0%, ratio of DEGs/all unigenes in the GO term). On the “molecular function” term, following “lysozyme activity” (75.0%) was “endodeoxyribonuclease activity, producing 5'-phosphomonoesters” (50.0%), “alcohol oxidase activity” (42.9%), and “oxidoreductase activity, acting on the CH-NH_2_ group of donors” (25.8%) in S1/S2 up analysis (Figure 4C). The GO terms “cellular component” involved in the DEGs were shown in Supplementary Figure 1. In conclusion, the unigenes that were upregulated in the AP_S1 of *A. polytricha* were enriched in the synthetic pathways and metabolic pathways, which were consistent with the high biomass of AP_S1.

**Figure 5:**
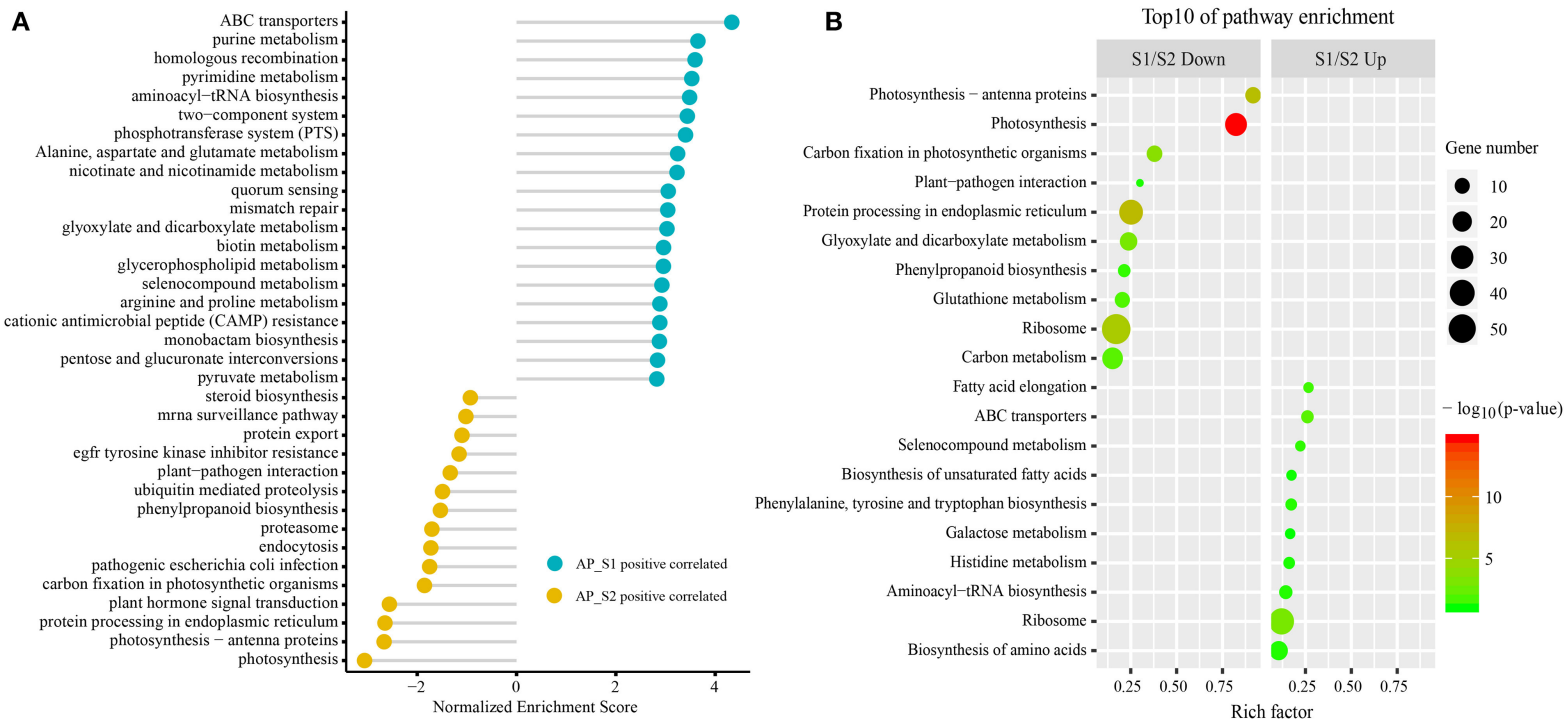
The Kyoto Encyclopedia of Genes and Genomes (KEGG) pathway enrichment analysis between the two different harvesting periods of *A. polytricha*. **(A)** The KEGG pathway enrichment analysis by the GSEA of all the unigenes. **(B)** The top 10 KEGG enrichment pathways of the downregulated (left) and upregulated (right) unigenes, respectively.

To gain the exhaustive gene function information, all the unigenes were analyzed based on the canonical pathways in the KEGG database by the GSEA (Figure 5A; Supplementary Table 8). Unigenes were clustered in “ABC transporters,” “homologous recombination,” “phosphotransferase system (PTS),” and “alanine, aspartate, and glutamate metabolism” in AP_S1 and “plant hormone signal transduction,” “Parkinson's disease,” “photosynthesis-antenna proteins,” and “plant–pathogen interaction” in AP_S2. Furthermore, classification of the two-fold changed unigenes among the KEGG pathways was also performed (the top 10 KEGG pathways by *p*-value) (Figure 5B; Supplementary Table 9). As shown in Figure 5B, the majority of the most abundant unigenes in AP_S1 was associated with the basic metabolism pathway such as “fatty acid elongation” (26.7%), “ABC transporters” (26.1%), “biosynthesis of unsaturated fatty acids” (17.4%), “phenylalanine, tyrosine, and tryptophan biosynthesis” (17.2%), “histidine metabolism” (16.1%), “aminoacyl-tRNA biosynthesis” (14.3%), “ribosome” (12.0%), and “biosynthesis of amino acids” (10.4%). S1/S2 down analysis showed that the downregulated unigenes in AP_S1 were focused on “photosynthesis-antenna proteins” (91.7%), “photosynthesis” (82.4%), “carbon fixation in the photosynthetic organisms” (37.9%), and “plant–pathogen interaction” (30.0%).

### Upregulated Unigenes of AP_S1 in the Amino Acid Synthesis and Energy Metabolism Pathways

**Figure 6:**
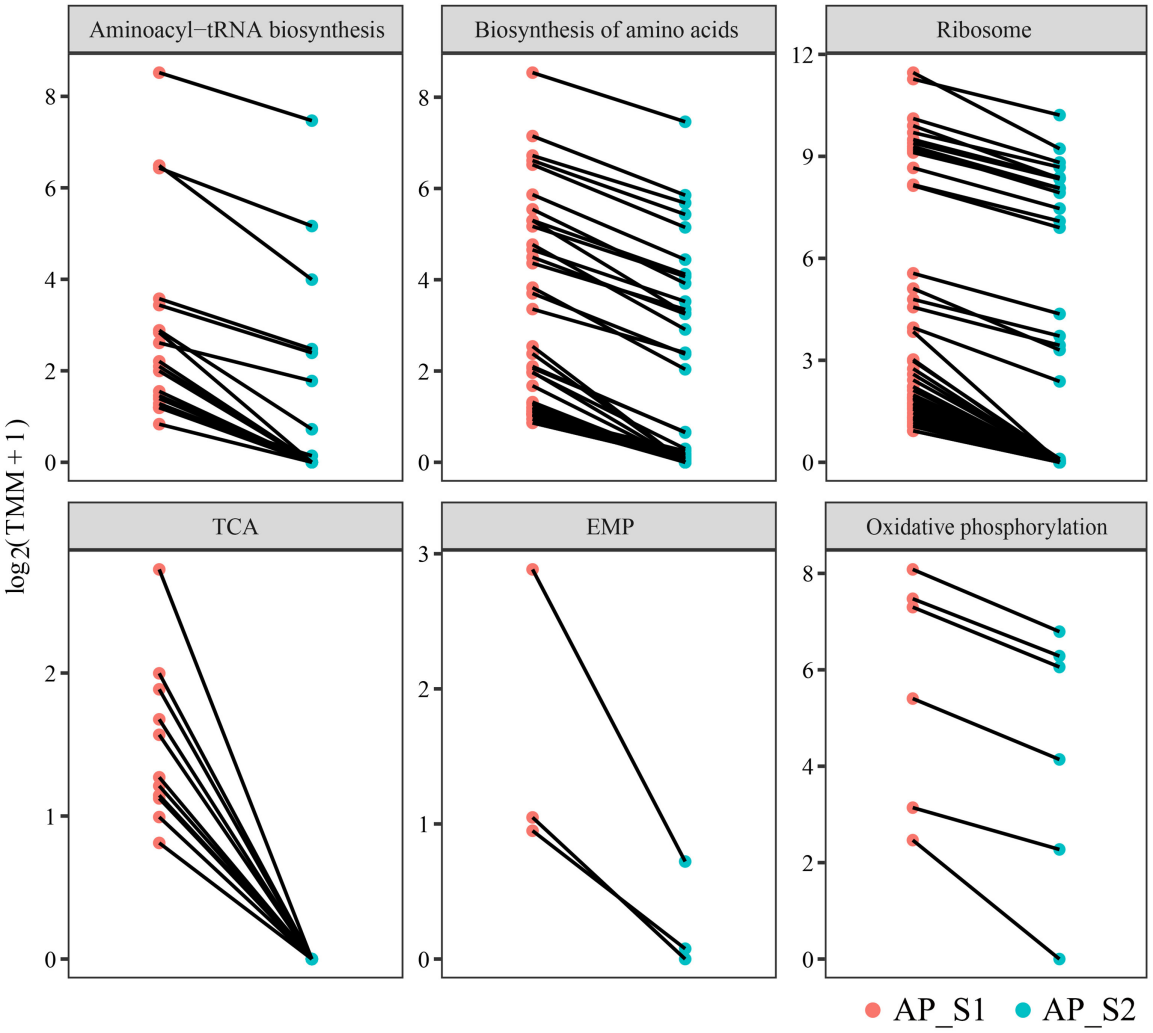
Differentially expressed level of the DEGs involved in the amino acid synthesis and energy metabolism pathways among the two different harvesting periods of *A. polytricha*.

To further investigate the difference of the nutritional properties between AP_S1 and AP_S2, the unigenes involved in the amino acid synthesis and energy metabolism pathways were selected to excavate deeply the regulatory mechanism. The result of the KEGG pathway analysis demonstrated that more DEGs were involved in the amino acid metabolism such as “aminoacyl-tRNA biosynthesis” (20 unigenes), “biosynthesis of amino acids” (43 unigenes), and “ribosome” (50 unigenes) (Figures 5B, 6; Supplementary Table 10). The powerful function of ribosome could provide the site for protein synthesis, which can improve the nutrition of *A. polytricha*. The upregulated energy metabolism pathways such as “tricarboxylic acid cycle” (11 unigenes), “glycolytic pathway” (3 unigenes), and “oxidative phosphorylation” (6 unigenes) would provide more energy for AP_S1 to build up more biomass. Moreover, a large number of DEGs upregulated in the fruiting body was related with the biosynthetic process of lipopolysaccharide, peptidoglycan, purine, lipid, pyrimidine nucleotide, porphyrin-containing compound, pyridoxine, and phospholipid.

### Real-Time Reverse Transcription-PCR Validation of the Differentially Expressed Gene Results

**Figure 7:**
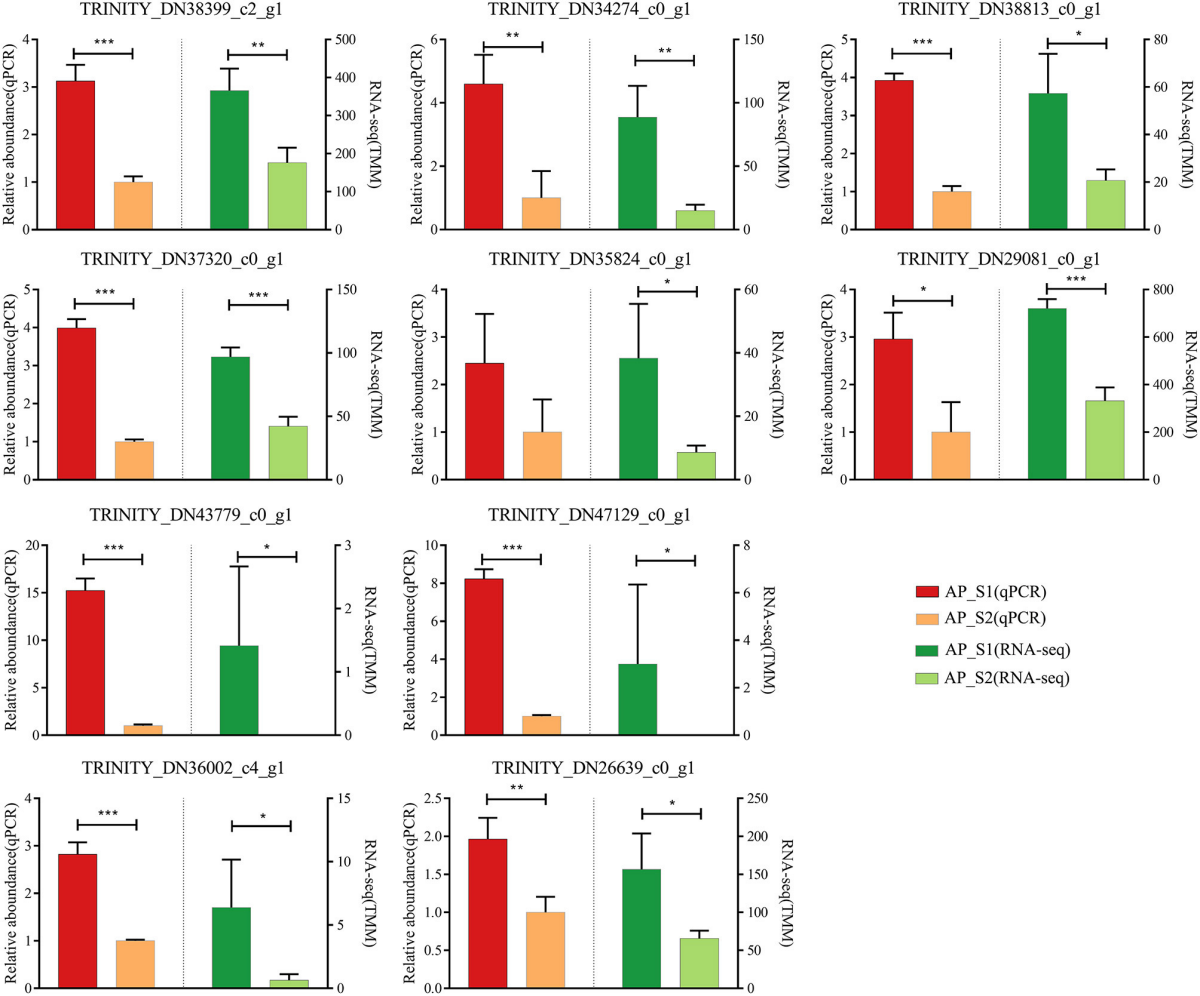
Verification of the 10 selected DEGs in RNA sequencing (RNA-Seq) by the quantitative reverse transcriptase-PCR (qRT-PCR). Left half of bar plot shows the expression levels determined by the qRT-PCR, whereas the right half indicates the results from the RNA-seq. *P < 0.05, **P <0.01, ***P < 0.001.

To evaluate the validity of the Illumina analysis, real-time RT-PCR was conducted to confirm the expression levels of 10 selected DEGs with the TRINITY_DN35671_c1_g2 (*actin*) as the reference gene (Figure 7; Supplementary Table 11). The 10 selected DEGs were involved in the amino acid and protein synthesis pathways [TRINITY_DN38399_c2_g1 (*LysRS*), TRINITY_DN34274_c0_g1 (*RecE*), TRINITY_DN37320_c0_g1 (*Rpe*), TRINITY_DN35824_c0_g1 (*Mrl*), TRINITY_DN38813_c0_g1 (*Pdh*), and TRINITY_DN29081_c0_g1 (*RPL37*)] and the energy metabolism pathways [TRINITY_DN43779_c0_g1 (*AcnA*), TRINITY_DN47129_c0_g1 (*Icd*), TRINITY_DN36002_c4_g1 (*HypF*), and TRINITY_DN26639_c0_g1 (*Uqcrc2*)]. The results showed that all of the 10 unigenes expression profiles were increased in AP_S1 compared with AP_S2, which showed that the expression profile of DEGs was in agreement with the RNA-seq analysis.

## Discussion

Traditionally, mushrooms have been used in the medical field as a source to develop the new drugs because of the presence of a large number of bioactive compounds including polysaccharides, triterpenes, nucleosides, and glycopeptides (19). Among the important metabolites, much attention has been paid to the polysaccharides due to their attractive bioactivities such as regulating immune responses (20), hepatoprotective activities (21), and tumor (22). Triterpenoids and their related compounds are fairly common among the mushroom metabolites, which are involved in anticancer (23), antibacterial (24), and antihyperlipidemia activities (25). However, there are few studies on the relationship between the transcription and development of edible mushroom. At present, the sequencing of genome or transcriptome of various mushrooms has been reported, for example, *Schizophyllum commune* (26), *Coprinopsis cinerea* (27), *Lentinula edodes* (28), *Ganoderma lucidum* (29), *Agrocybe aegerita* (30), *Cordyceps militaris* (31). As a nutritional and medical mushroom, *A. polytricha* has attracted considerable attention because of its biological active components such as trace elements, lectins, and terpenoids (32–37). The cultivation of *A. polytricha* is seasonal, which can be harvested several times in one cultivated bag every year. More importantly, there are big differences of the nutrition and quality among the two different harvesting periods of *A. polytricha*, but little transcriptome information is currently available.

High-throughput sequencing technologies, such as transcriptomics, genomics, proteomics, and metabolomics, make it possible to use a system biology approach to assess the changes in the plant developmental molecular biology to identify the key developmental genes and to investigate the growth regularity. As most complex developmental process, it is of importance to analyze the fruiting body formation in the mushrooms (38). In this study, we investigated the discrepancy between AP_S1 (the stage for the first harvesting period) and AP_S2 (the stage for the third harvesting period) by using statistical analyses including character, nutrition, quality, and transcriptional changes. This study indicated that the fruiting body of AP_S1 had better shape and higher biomass, area, and auricle thickness, which contributed to more growth advantage compared to AP_S2 (Figure 1). Moreover, the comparison of nutrients, mineral elements, and contents of amino acids between AP_S1 and AP_S2 suggested that some element contents, such as protein and calcium, were apparently higher in AP_S1 compared to AP_S2 (Supplementary Table 1). The most species of the amino acids and total amino acid content in AP_S1 were higher compared to AP_S2, except Met, Thr, Asp, and Phe (Supplementary Table 2). However, the fat content of AP_S1 was lower compared to AP_S2, fully testifying that edible mushroom is a kind of high-protein and low-fat food. Owing to the fact that AP_S1 had a good appearance, good nutrition, and a good taste, the acceptability of AP_S1 was apparently higher compared to AP_S2 (Figure 1E).

In this study, the comparison of transcriptome libraries was carried out to determine the unigene expression variation between AP_S1 and AP_S2. Both *p* ≤ 0.05 and absolute value of log_2_ratio ≥ 1 were regarded as the threshold to evaluate the significance of unigene expression levels. To investigate the biological and molecular functions, an in-depth analysis on all the unigenes and the significantly DEGs (Supplementary Table 5) was conducted. The GO annotation of all the unigenes showed that the minority of DEGs was found in the clusters of virion part, metallochaperone activity, locomotion, protein-binding transcription factor, receptor, immune system process, cell junction, nucleoid, symplast, virion, positive regulation of biological process, and reproductive process (Figure 4). These findings suggested that the functions of DEGs belonging to these groups may have nothing to do with the fruiting body development. Functional enrichment analysis of the GO demonstrated that the unigenes involved in “biosynthetic process,” “metabolism- and synthesis-related enzyme activity,” and “DNA-associated process and enzyme activity” were dominant in AP_S1 and the terms of “photosynthesis,” “response,” and “signaling pathway” were dominant in AP_S2 (Figures 4A,B; Supplementary Table 6). The metabolic processes such as “small molecule biosynthetic process,” “secondary metabolic process,” and “terpenoid biosynthetic process” and the molecular functions such as “catalytic activity,” “oxidoreductase activity,” “ligase activity,” and “active transmembrane transporter activity” were enhanced in AP_S1, which satisfied the growth accumulation needs of AP_S1 (Figure 4C). In comparison to AP_S1, the most upregulated DEGs were sorted into the less effective aspects for the growth and development such as “photosynthesis,” “response,” and “signaling pathways.” These results suggested that in the fruiting body development process, the biosynthesis, metabolism, and assembly showed relatively more active.

The KEGG pathway analysis showed that more upregulated DEGs of “AP_S1” participated in the amino acid and protein metabolism such as “ribosome,” “phenylalanine, tyrosine, and tryptophan biosynthesis,” “aminoacyl-tRNA biosynthesis,” and “biosynthesis of amino acids” (Figure 5). The “ribosome” was also enhanced to provide a place for the protein synthesis and, in turn, the protein synthesis further promoted the assembling of ribosome. These results indicated that the amino acid metabolism, including the metabolism of tryptophan and tyrosine, was more active in the fruiting body development, consistent with a previous study (11). Both “fructose and mannose metabolism” and “amino sugar and nucleotide sugar metabolism” pathways were involved in the biosynthesis of polysaccharides, making *A. polytricha* high polysaccharide contents (Supplementary Table 8). In addition, the steroid hormone biosynthesis pathway was highly expressed in AP_S1, whereas the photosynthesis was highly expressed in AP_S2. This discrepancy implied that the synthesis of metabolites in AP_S1 and AP_S2 was different, therefore producing the different nutrients in these two harvesting periods of *A. polytricha*. In addition, the pathways of “tricarboxylic acid cycle,” “glycolytic pathway,” and “oxidative phosphorylation” were enhanced in AP_S1 compared to AP_S2, which provided the energy for AP_S1 to maintain the synthesis and metabolism. To evaluate the validity of Illumina analysis, real-time RT-PCR was conducted to confirm the expression levels of 10 selected DEGs. The results showed that the expressions of 10 selected DEGs involved in the amino acid and protein synthesis pathways and the energy metabolism pathways were enhanced in AP_S1 compared to AP_S2, consistent with the Illumina analysis.

To the best of our knowledge, this is the first study to investigate the differences between AP_S1 and AP_S2 on the basis of *de novo* transcriptome sequencing. The results demonstrated that more DEGs upregulated in AP_S1 were involved in the synthesis and metabolism, especially amino acid, protein, and metabolism. Moreover, the strengthened tricarboxylic acid cycle can provide more energy for the accumulation of biomass and nutrient for AP_S1. In summary, this study presented the essential reference information for further study regarding the developmental and genetic characteristics of *A. polytricha*.

## Data Availability Statement

The datasets presented in this study can be found in online repositories. The names of the repository/repositories and accession number(s) can be found in the article/Supplementary Material.

## Author Contributions

WW led and coordinated the project. WW and YW carried out the bioinformatics analyses and wrote the manuscript. SY and ZG revised the manuscript. All authors read and agreed with the final manuscript. FJ is the corresponding author and responsible for all contact and correspondence.

## Funding

This study was funded by National Natural Science Foundation of China (31801608 and 32002329), Natural Science Foundation of Shandong Province (ZR2020QC197), and Innovative Engineering project of Shandong Academy of Agricultural Sciences (CXGC2021A15).

## Conflict of Interest

The authors declare that the research was conducted in the absence of any commercial or financial relationships that could be construed as a potential conflict of interest.

## Publisher's Note

All claims expressed in this article are solely those of the authors and do not necessarily represent those of their affiliated organizations, or those of the publisher, the editors and the reviewers. Any product that may be evaluated in this article, or claim that may be made by its manufacturer, is not guaranteed or endorsed by the publisher.

## Supplementary Material

Supplementary Figure 1 The GO terms cellular component involved in the DEGs were shown.

Supplementary Table 1 Quality comparison between the two different harvesting periods of *Auricularia polytricha (A. polytricha)*.

Supplementary Table 2 Contents of the amino acids in the two different harvesting periods of *A. polytricha* (%).

Supplementary Table 3 High-throughput sequencing analysis and quality of the Illumina sequencing of *A. polytricha* transcriptome.

Supplementary Table 4 Correlation analysis of AP_S1_1, AP_S1_2, AP_S1_3, AP_S2_1, AP_S2_2, and AP_S2_3.

Supplementary Table 5 Two-fold differentially expressed gene annotation summary.

Supplementary Table 6 The Gene Ontology (GO) analysis of all the unigenes by the gene set enrichment analysis (GSEA).

Supplementary Table 7 The GO analysis of the two-fold differentially expressed genes.

Supplementary Table 8 The Kyoto Encyclopedia of Genes and Genomes (KEGG) pathway analysis of all the unigenes by the GSEA.

Supplementary Table 9 The KEGG analysis of the two-fold differentially expressed genes.

Supplementary Table 10 Upregulated unigenes of “AP_S1” in the amino acid synthesis and energy metabolism pathways.

Supplementary Table 11 Primers used in this study.
