# Supplementary figures and images for: Comparison of the Nutritional Properties and Transcriptome Profiling Between the Two Different Harvesting Periods of Auricularia polytricha
Source: Front Nutr. 2021 Oct 26;8:771757. doi: 10.3389/fnut.2021.771757 (PMC8576271; doi:10.3389/fnut.2021.771757)

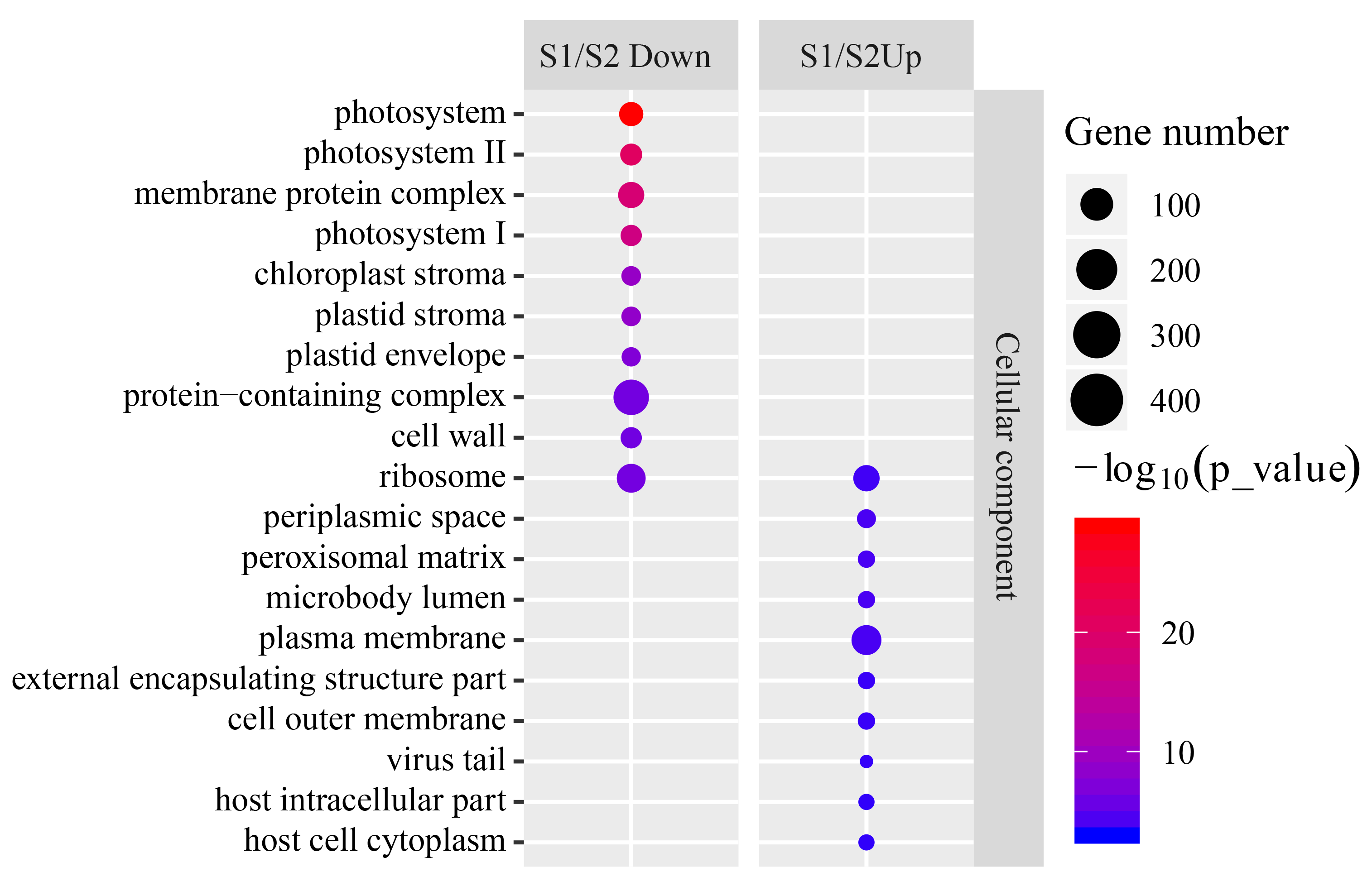

Supplement: Supplementary Figure 1 — The GO terms cellular component involved in the DEGs were shown. [file Image_1.TIF]
