# Supplementary material for: Comparison of the Nutritional Properties and Transcriptome Profiling Between the Two Different Harvesting Periods of Auricularia polytricha
Source: Front Nutr. 2021 Oct 26;8:771757. doi: 10.3389/fnut.2021.771757 (PMC8576271; doi:10.3389/fnut.2021.771757)
Supplement: Supplementary Table 1 — Quality comparison between the two different harvesting periods of Auricularia polytricha (A. polytricha). [file Table_1.docx]

Table S1 Quality comparison between two different harvesting periods of *A. polytricha*

| Sample name | Fe (mg/kg) | Ca (mg/kg) | Mg (mg/kg) | Cu (mg/kg) | Zn (mg/kg) | Protein (%) | Fat (%) | Fibre (%) |
| --- | --- | --- | --- | --- | --- | --- | --- | --- |
| AP_S1 | 43.6±4.2 | 1600±135 | 1630±113 | 1.38±0.08 | 7.05±0.53 | 12.0±1.3 | 0.2±0.07 | 19.5±1.1 |
| AP_S2 | 67.0±6.2 | 1230±102 | 1660±175 | 1.34±0.07 | 8.14±0.69 | 10.8±1.0 | 0.8±0.14 | 18.6±2.0 |
