# Supplementary material for: Comparison of the Nutritional Properties and Transcriptome Profiling Between the Two Different Harvesting Periods of Auricularia polytricha
Source: Front Nutr. 2021 Oct 26;8:771757. doi: 10.3389/fnut.2021.771757 (PMC8576271; doi:10.3389/fnut.2021.771757)
Supplement: Supplementary Table 2 — Contents of the amino acids in the two different harvesting periods of A. polytricha (%). [file Table_2.docx]

Table S2 Contents of amino acids in two different harvesting periods of *A. polytricha* (%)

| Amino acid  composition | Content | | Amino acid  composition | Content | |
| --- | --- | --- | --- | --- | --- |
|  | AP_S1 | AP_S2 |  | AP_S1 | AP_S2 |
| Lys | 0.47±0.06 | 0.38±0.04 | Thr | 0.26±0.05 | 0.41±0.03 |
| Tyr | 0.11±0.03 | 0.06±0.01 | Glu | 0.92±0.10 | 0.64±0.07 |
| Ile | 0.34±0.07 | 0.25±0.04 | Val | 0.57±0.04 | 0.45±0.04 |
| Cys | 0.21±0.08 | 0.17±0.02 | Asp | 0.45±0.10 | 0.71±0.08 |
| Leu | 0.81±0.15 | 0.6±0.10 | Ser | 0.39±0.05 | 0.35±0.03 |
| Pro | 0.65±0.11 | 0.47±0.05 | His | 1.76±0.04 | 1.59±0.10 |
| Met | 0.20±0.06 | 0.50±0.08 | Phe | 2.66±0.09 | 2.93±0.09 |
| Ala | 0.74±0.08 | 0.54±0.09 | Gly | 0.46±0.02 | 0.34±0.03 |
| Arg | 0.51±0.07 | 0.35±0.04 | Totle | 11..5103 | 10.7403 |
| Trp | 0.0005±0.0003 | 0.0003±0.0001 |  |  |  |
