# Supplementary material for: Comparison of the Nutritional Properties and Transcriptome Profiling Between the Two Different Harvesting Periods of Auricularia polytricha
Source: Front Nutr. 2021 Oct 26;8:771757. doi: 10.3389/fnut.2021.771757 (PMC8576271; doi:10.3389/fnut.2021.771757)
Supplement: Supplementary Table 3 — High-throughput sequencing analysis and quality of the Illumina sequencing of A. polytricha transcriptome. [file Table_3.docx]

Table S3 Throughput and quality of Illumina sequencing of *A. polytricha* transcriptome

| Sample name | Raw  reads | Clean  reads | Clean bases | Valid (%) | Q30 (%) | GC content (%) | Unigenes |
| --- | --- | --- | --- | --- | --- | --- | --- |
| AP_S1_1 | 68861618 | 60520044 | 9.1G | 87.89 | 91.22 | 62.23 | 38414 |
| AP_S1_2 | 65565390 | 57559938 | 8.6G | 87.79 | 91.33 | 62.39 | 37529 |
| AP_S1_3 | 69712364 | 60293772 | 9.0G | 86.49 | 90.91 | 62.82 | 40818 |
| AP_S2_1 | 109963214 | 101848076 | 15.3G | 92.62 | 96.66 | 61.67 | 49176 |
| AP_S2_2 | 107793618 | 101581998 | 15.2G | 94.24 | 96.9 | 62.15 | 51396 |
| AP_S2_3 | 109252364 | 102712900 | 15.4G | 94.01 | 96.85 | 61.53 | 51433 |
